# Ovarian Stainology: Database of evidence-based immunohistochemical antigen expression in ovarian tumors

**DOI:** 10.1038/s41597-026-07776-1

**Published:** 2026-06-30

**Authors:** Jan H. Gruszczyński, Kacper Trębacz, Emilian Małek, Michał J. Dopierała, Aleksander Gostyński-Glina, Patryk Kraiński, Hanna Pelant, Miłosz Kadziński

**Affiliations:** 1 Institute of Computing Science, Poznan University of Technology, Poznan, Poland; 2 Faculty of Medicine, Poznan University of Medical Sciences, Poznan, Poland; 3 Faculty of Mathematics and Information Science, Warsaw University of Technology, Warsaw, Poland; 4 Department of Pathology and Wildlife Diseases, Swedish Veterinary Agency, Uppsala, Sweden; 5 Department of Oncological Pathology, University Clinical Hospital, Poznań University of Medical Sciences, Poznan, Poland; 6 Faculty of Computing and Telecommunications, Poznan University of Technology, Poznan, Poland; 7 Department of Clinical Pathology and Immunology, Poznan University of Medical Sciences, Poznan, Poland

## Abstract

Immunohistochemical (IHC) profiling is essential for ovarian tumor subtyping, but comprehensive data on antigen and protein expression frequencies is fragmented across the literature. We developed a structured, harmonized database of IHC expression profiles in ovarian tumors extracted from published studies. The dataset was constructed using large language models to identify and extract relevant data from abstracts and tables from systematically retrieved publications. Screening of 5,961 studies identified 100,149 raw tumor-stain pairs, which were processed through a multi-step curation pipeline to filter, standardize, and harmonize the records. The final dataset comprises 12,212 tumor–stain frequency records derived from 1,450 studies, with all tumor nomenclature standardized to the 2020 WHO Classification of tumors. Records include details such as expression type, total sample counts, expression percentages, subcellular localization and expression distribution. The resource provides a consolidated, evidence-based reference to support research, analyses, and the development of computational models in diagnostic pathology.

## Background & Summary

The diagnosis of ovarian tumors relies heavily on immunohistochemical (IHC) profiling to distinguish histological subtypes that are known to correlate with prognosis^1^ and/or specific therapeutic strategies^2^. Immunophenotyping aids this distinction by evaluating the expression of specific proteins (antigens of proteins) within tumor tissue samples. Subtype and origin of a neoplasm are commonly associated with a characteristic immunophenotypic profile, defined by expected expression features such as expression type (e.g., positive, negative, over-expression, or loss of expression), subcellular localization (e.g., nuclear, cytoplasmic, or membranous), and many other context-dependent variations across a panel of immunostains targeting specific protein antigens. Therefore, accurate interpretation of immunostain panel results - and identification of the most likely underlying tumor subtype - requires knowledge of the frequency with which specific expression features of proteins occur across different tumor subtypes. Although described immunophenotypic data is central to diagnosis accuracy, large-scale data mapping the immunophenotypic landscape of ovarian neoplasms remains limited.

Currently, the primary sources of immunophenotypic data include online resources such as the *WHO Classification of tumors* (Blue Book series, 2020)^3^ and the Ovary section of PathologyOutlines^4^, as well as the broader body of published literature on which these resources are based. Both the *WHO Blue Book* and PathologyOutlines are widely used and represent authoritative, high-quality resources that provide accessible summaries of immunostain expression across tumor types. However, they remain limited in scope, covering only a subset of commonly used immunostains and, in most cases, without frequency of specific pattern expression. The published literature, though extensive, is fragmented across numerous studies with variable aims, methodologies, different reporting formats, and conflicting results, making interpretation challenging and time-consuming.

Expression data reported in the literature is highly heterogeneous: terminology is inconsistent; expression data is often stratified by non-standard, study-specific categories (e.g., tumor grade, patient age, biomarker values); IHC expression results are reported using vastly varying scoring systems. This heterogeneity makes cross-study comparison, aggregation, and interpretation of expression frequencies impractical without a structured and harmonized resource.

To address these limitations, we developed the Ovarian Stainology database—a structured, openly accessible repository of IHC expression profiles in ovarian neoplasms. The dataset provides a comprehensive, evidence-based summary of IHC findings aggregated from the literature. The final harmonized dataset comprises 12,212 tumor–stain frequency records derived from 1,450 published studies, curated from 100,149 pairs gathered from 5,961 screened studies. Records in the database include features such as expression type (e.g., positive, loss, over-expression), intensity, distribution, total sample size, percentage of samples with a given expression, and subcellular localization. All tumor nomenclature has been harmonized according to the 2020 WHO Classification of tumors^3^.

The database was constructed through a systematic identification of relevant publications in PubMed. Large language models (LLMs) were employed to screen abstracts and table-like figures for IHC-related data and to perform standardized data extraction according to a predefined schema. A multi-step post-processing pipeline was subsequently applied to filter, standardize, and harmonize the extracted information, yielding a consistent and analysis-ready dataset.

The database is designed to facilitate potential reuse in research, including meta-analyses of protein expression, computational model development, and providing an aggregated reference for protein expression frequencies across various ovarian tumor subtypes. With continued refinement and clinical validation, the resource may also serve as a foundation for future decision-support tools in ovarian diagnostic pathology, for instance, to inform the selection of IHC panels or aid in results interpretation. The full dataset is available for download. This includes both the processed and harmonized dataset (designed for direct analysis) and the raw, unharmonized data as extracted from the literature, providing transparency and enabling researchers to apply their own custom curation methods.

## Methods

The **Stainology database** was constructed through systematic retrieval, extraction, and curation of published data on IHC results in ovarian tumors. The process involved (i) literature retrieval, (ii) extraction of abstracts and table-like figures, (iii) identification of relevant IHC content, (iv) structured extraction of IHC data, (v) tumor nomenclature assignment according to the *WHO Classification of tumors* (Blue Book series, 2020 edition)^3^, and (vi) data curation and harmonization.

### Journal articles retrieval

The input data consisted of journal articles indexed in the PubMed database, retrieved using the PubMed query utility. Retrieval partially adapted methods from the Preferred Reporting Items for Systematic Reviews and Meta-Analyses (PRISMA) guidelines^5^ and the Cochrane Handbook for Systematic Reviews of Interventions^6^.

The retrieval aimed to identify studies meeting the following inclusion criteria:

1. They involved ovarian tumors.
2. They reported, at least partially, results from IHC staining applied to these tumors.
3. They were published in the period between 1 January 1997 and 1 June 2025, and were written in English. (Year 1997 was chosen to align with the earliest references cited in PathologyOutlines^4^, in order to include important older studies involving widely used immunostains, which are not the subject of newer studies.)

Targeted PubMed queries were constructed for each immunostain individually. We began with a list of 154 immunostains routinely used at institutions affiliated with the pathologist co-authors of this study. In a second search iteration, we expanded this list to include 895 additional immunostains that were identified in the initially retrieved articles. For every stain, a dedicated query was generated that incorporated all common naming variants (e.g., for PAX8: “PAX-8,” “pax-8,” “PAX8,” “pax8”), which were compiled into a single OR-clause.

The disease component of the query combined the official PubMed Medical Subject Heading (MeSH, used for topic-indexing of articles) term “Ovarian Neoplasms” combined using an OR statement with a set of ovarian tumor–related free-text synonyms (e.g., “ovarian cancer,” “ovarian carcinoma,” “ovarian tumor,” “ovarian neoplasm,” “ovarian malignancy,” “ovarian malignancies,” “ovarian adenocarcinoma,” “ovarian epithelial tumor,” “ovarian mass,” “ovarian cystadenocarcinoma”), searched in titles and abstracts.

Immunohistochemistry was captured both through its MeSH term “Immunohistochemistry” and free-text variations (e.g., “immunohistochemistry,” “immuno-histochemistry,” “IHC,” “IHC staining,” “immunostaining”).

Additionally, the query excluded the following studies:

- Studies involving animals (by the use of the MeSH term “Animals”), because the study focus was on human ovarian tumors.
- Studies classified as Case Reports (by the use of Publication Type and case report keywords in the title or abstract), to avoid single-patient data.
- Studies involving diagnostic errors and reclassifications (by the use of specific keywords appearing in the title or abstract and MeSH term “Diagnostic Errors”), as it would be difficult to distinguish between primary and reclassified frequency records.

The exact full query syntax and both lists of stains are available in the project’s code repository, referenced in the *Code Availability* section of this article.

### Extraction of abstracts and table-like figures

For each retrieved study, all available metadata, including abstracts, was obtained using the PubMed API via the Bio.Entrez module in the Biopython Python package^7^. When possible, full-text PDF files were acquired either through the OpenAlex^8^ API or manually via institutional library access available to the study authors.

Detection of table-like figures within the PDFs was performed using the *“microsoft/table-transformer-detection”* deep learning model^9^, shared through the Hugging Face model hub and was accessed via Transformers Python Package. The model was applied to every page of each PDF to identify regions corresponding to tabular content. Detected regions were cropped with additional vertical and horizontal padding to (possibly) include associated captions or legends where present.

### Extraction of relevant IHC data

Identification of relevant IHC content and structured data extraction was performed using an LLM approach. Specifically, the study employed Google’s Gemini 2.5 Pro (Vertex AI model gemini-2.5-pro, general audience (GA) release 17 June 2025) model. We selected this model based on the state-of-the-art performance metrics reported in the official *Gemini 2.5* technical report^10^ and model card^11^, as well as its position as the top-ranking model in the Vision Arena and Text Arena standings on the community-curated LMSYS Chatbot Arena^12^ at the time of writing (accessed 20 August 2025).

### Abstract extraction

For abstracts, a single-step approach was used in which the model was prompted with the study title and abstract for context, and detailed instructions specifying the target data fields to be extracted supplemented with a set of 24 standardization rules specifying how extraction should be performed. The purpose of the rules was to increase the consistency and standardize extracted outputs across studies. If a given abstract did not contain the specified data the LLM was instructed to return a note stating “Bad Abstract” indicating irrelevancy to the study.

### Table-like figures extraction

For table-like figures, a two-step prompting chain was employed. In the first step, that identified relevancy of the table, the LLM was asked to extract tumor names, IHC stains, stain thresholds, and stain intensities from the table-like figure. In this step, the LLM was also instructed to discard tables which met following discard criteria:

- The figure contains no IHC data or is based solely on genetics data.
- The figure does not contain names of neoplasms (cancers/tumors).
- The figure is focused on individual patient cases.
- The figure does not contain case distribution data (e.g., counts or percentages of patients/samples categorized by a feature).
- The figure reports diagnostic metrics (e.g., sensitivity, specificity, positive/negative predictive values).

The discard criteria were introduced to exclude figures that did not contain IHC data, could not be consistently parsed into structured IHC records or that contained non-comparable information (e.g., single-case data or diagnostic metrics).

The second step was executed only if the first step identified at least one neoplasm and one stain, and the discard criteria were not met. In the second step, the model was again provided with the table-like figure, along with the study title and abstract for context, and was given detailed instructions specifying the target data fields to extract along with the same set of 24 rules used during the abstract’s extraction. Also similarly to the abstracts, if a given table-like figure did not contain the specified data the LLM was instructed to return a note stating “Bad Table” indicating irrelevancy to the study. Consequently, each table-like figure could be rejected during either the first or second step.

### Target data fields and subdivision

For both the abstract and table-like figures, the model was prompted to extract the same set of target fields, which correspond exactly to the database fields specified in Table 1 of the *Data Records* section of this study. The list of fields was designed to accommodate the widest possible range of IHC result reporting styles.

To accommodate studies that subdivided the IHC results based on one or more of the following criteria: expression score, expression threshold, expression intensity, distinct values for said fields were preserved as separate rows. For example, the following would be extracted and stored as individual entries: *tumor – stain – positive expression – x % of patients – score 1+* and *tumor – stain – positive expression – y % of patients – score 2+*. Depending on the study, the subdivided groups could or could not be overlapping (consecutive groups don’t contain cases from previous groups); in most studies, a non-overlapping approach is taken. Similarly, if a study subdivided the expression data by expression type, pattern, or expression distribution, each entry was kept as a separate row.

### Output format and prompting structure

For both abstracts and table-like figures, the model was further instructed to omit any fields other than tumor name, stain, and expression type unless this information was explicitly present in the source material. Additionally, the model was instructed to return the result as a list of JSON-like dictionaries. Each dictionary represented a single row (tumor-stain pair), with its keys marking the columns and the corresponding values representing the data for that row. Prompting followed Google’s best practice guidelines^13^, using the structure: role, aim, input, example (for abstracts only), and expected output format.

All prompt templates, the 24 standardization rules, and the scripts used to execute the extraction chains are publicly available in the project’s code repository, referenced in the *Code Availability* section of this article.

### Tumor nomenclature assignment according to the WHO

To enable the aggregation of IHC expression data across studies, it was necessary to standardize the nomenclature of tumor subtypes reported in the literature. The *WHO Classification of tumors* (Blue Book series, 2020 edition)^3^ was used as the gold standard reference for the tumor subtype hierarchy. LLMs were employed to assign the names or acronyms, previously extracted during the extraction stage (*Extraction of relevant IHC data*), to their respective WHO tumor classes or categories.

To validate this method, a test set comprising 535 extracted tumor names from 181 studies was created. The terms were selected from the set of extracted tumor-stain pairs, provided they were associated with a non-empty expression percentage field. Two certified pathologists manually mapped these terms to the WHO classification (tumor classes, Category Level 1, or Level 2) to create a ground truth dataset. Two additional custom categories were appended: “Artificial Taxon” (for entries not recognized by the WHO classification) and “Not Ovarian” (for non-ovarian entities).

Pathologists were instructed to adhere to the following rules: classifications should be assigned at the highest possible level of specificity, provided there is no uncertainty. When in doubt, the annotator should first attempt to assign a tumor class, followed by Category Level 1, and then Category Level 2. If uncertainty remained, or if there was no clear mapping from an older WHO classification, the term was assigned to the “Artificial Taxon” category.

LLMs were then prompted to perform the same classification task following the identical mapping rules. Both the pathologists and the LLMs were provided with the following contextual information: study title, publication date, abstract, and all fields relevant to the task (*tumor*, *Group*, *Group Category*, and *Notes*). Access to the full study content was not permitted. LLM-based classification was performed independently for each study, using prompts that included all tumor names extracted during the Data Extraction stage (from both the abstract and any detected table-like figures), along with the contextual information specified above and the appended Ovarian WHO classification tree (tumor classes were provided in the prompt along with their Blue Book definitions). Consequently, the assigned WHO classification for each term was study-specific and context-dependent. To illustrate, if an article used a general term like “carcinoma” but its abstract explicitly restricted the scope to high-grade serous carcinomas, the term was classified as such. This context-based approach allowed identical general terms to be classified distinctly based on the explicit description within each source, increasing the amount of preserved data.

Prompting followed the same guidelines as described above (*Extraction of abstracts and table-like figures*). The complete prompt template is provided in the project’s code repository, referenced in the *Code Availability* section of this article, along with scripts used to execute the classification.

The LLMs assessed included Google’s *Gemini 2.5 Pro* (Vertex AI model gemini-2.5-pro, GA release: 17 June 2025), *Gemini 2.5 Flash* (Vertex AI model gemini-2.5-flash, GA release: 17 June 2025), OpenAI’s *GPT-5* (OpenAI API model gpt-5, GA release: 7 August 2025), and *GPT-5-mini* (OpenAI API model gpt-5-mini, GA release: 7 August 2025). Model selection was guided by top standings in the LMSYS Chatbot Arena leaderboard^12^ (accessed 20 August 2025). The performance of each model was evaluated against the pathologist-annotated test set to select the optimal model for final classification (see *Validation of LLM-based WHO nomenclature assignment*). Based on this evaluation, Gemini 2.5 Flash was selected to classify all remaining unmapped tumor names in the database.

### Data curation and harmonization

The primary challenge in analysing (through comparison, aggregation, and interpretation) IHC data from published literature is the significant variability and lack of standardization in how results are reported and formatted, as outlined in Background & Summary of this study.

To address this challenge, a multi-step post-processing curation pipeline was developed to filter, standardize, and harmonize the extracted raw data. The pipeline transforms the data into a harmonized form by unifying naming variations, removing study-specific stratification (except for tumor subtypes division), and aggregating disparate scoring/intensity/thresholds and distribution systems. Resulting in a dataset where, for a given tumor–stain pair in a study, frequency entries vary only by following features (columns): expression type, pattern/location, notes.

The pipeline was implemented as a set of modular classes, each responsible for a specific function, which are combined into a workflow (pipeline). The design allows the pipeline to be easily adapted or extended to meet specific analytical needs. Implementation of the pipeline and its classes can be found in the source code repository referenced in the *Code Availability* section of this article.

The pipeline consisted of the following steps:

### Step 1: Naming variation unification

All text cells in the table were cleaned using a standard function that converted all text to lowercase, removed extraneous whitespace, and retained only alphanumeric characters. Values in columns were unified by the use of automated handling of formatting variations as well by the use of manually prepared dictionaries of common variations, for instance: “cytoplasmic”: [‘cytoplasmic’, ‘cytoplasm’, ‘tumor cells, cytoplasmic’]. Specifically for stains, Greek-to-Latin character substitutions (e.g., β → beta) were performed.

### Step 2: Data filtering and correction

In this step, filtering and correction rules were applied:

- All entries corresponding to the following type of studies were dropped: reviews, systematic-reviews, meta-analyses. This was done to remove duplication of entries for a given study.
- Exclusion of all entries with following publication type marked in PubMed database: “editorial”, “letter”, “comment”, “news”, “interview”, “published erratum”, “retracted publication”, or “retraction of publication” were excluded. This was performed to exclude any sources that were not peer reviewed. We also manually validated that all remaining entries were marked with publication type corresponding to peer-reviewed works.
- Exclusion of rows where expression percentage was missing and could not be inferred based on total sample number and expression type sample number.
- Removal of rows with specific keywords (e.g., “amplification”, “sequencing” in the notes field) that indicated presence of data other than IHC.
- Applying a series of conditional overwrites, such as setting the expression type to “negative” when a score of “0” or “-” appeared, with the goal of standardization across studies and correction of errors stemming from the way data was presented or extracted by the LLM.

### Step 3: Selection of Histological Subtype Groupings

Many studies reported data stratified by clinical or pathological characteristics (e.g., age, tumor stage, grade). To enable comparability, we restricted the dataset to data grouped by histological subtype or data representing the whole study population. The following logic was applied:

- When a table or abstract contained a non-empty *group* column we applied manually curated inclusion and exclusion keyword lists to identify histology-based categories. Examples of inclusion terms included “histology”, “histotype”, and “classification”; examples of exclusion terms included “grade” and “age of”.
- If no histology-related group label was identified, we selected rows in which both the *group* and *group category* columns were null (empty).
- If neither null-column rows nor histology-matched groups were present, the pairs from that table or abstract were excluded.
- If multiple groups matched the inclusion keywords, a secondary list of histology-specific inclusion terms was applied to refine the selection; in some cases, multiple groups were retained.
- Entries for tables or abstracts where all rows had null values in both *group* and *group category* were retained, assuming they represented ungrouped study populations.

### Step 4: Addition of WHO classification

In this step, tumor name assignment according to *WHO Classification of tumors* (WHO Blue Book, 2020 edition)^3^ was added in a separate column. Section Tumor nomenclature assignment according to the WHO describes how the classification was performed. Additionally, rows corresponding to tumors classified as “*Not Ovarian*” or “*Artificial Taxon*” were dropped.

### Step 5: Aggregation of Subdivided Data

Many studies report IHC results by subdividing cases based on study-specific scoring systems (e.g., 1+, 2+, 3+), intensity levels (e.g., weak, moderate, strong), percentage thresholds (e.g., >20% positive cells) or expression distribution. To standardize these varied reporting formats into a single frequency rate, we developed a subdivision aggregation algorithm. An algorithm was applied individually to the data extracted from each table or abstract to consolidate these subdivided entries. The algorithm assumes that populations defined in the study for each expression score intensity or threshold are independent and not overlapping, and also that the sum of all patients across every expression levels sums up to the total sample count (for a given stain - tumor pair).

The algorithm processes the dataset to merge rows that represent subdivisions of a single experimental group. For entries of each table-like figure or abstract, the process goes as follows:

1. **Grouping:** Rows are first grouped by all experimental conditions, except for numeric fields (e.g. total sample count, expression percentage) and study-specific fields (score, intensity, threshold or expression distribution). Then following is performed:
  **If possible negative rows or null-rows are selected:** To prevent double-counting of patients, the algorithm first scans each group for a definitive “negative” or summary row. If a single row is explicitly typed as negative, or if a row contains null (empty) values for study-specific columns, this row is prioritized and retained. All other rows within that specific group are then discarded.
  **Aggregating Positive Subdivisions:** If no single negative or summary row is identified, the algorithm proceeds to merge all rows within the group, which are assumed to represent non-negative expression subdivisions. This merge operation consists of:
    i. The number of positive cases from each row is summed to yield a total number of positive cases.
    ii. The total cohort size is taken from the first row (after verifying it is consistent across all rows in the group).
    iii. The overall expression percentage is recalculated based on the new aggregated values.
  **Integrity Checks:** The algorithm includes several validation steps. Any table-like figure or abstract is flagged for exclusion if it contains following inconsistencies:
    i. Varying total sample count values for rows that should belong to the same cohort.
    ii. The final summed specific expression sample being greater than the total sample size, implying broken independence of subdivision population assumption.
    iii. Multiple ambiguous summary rows or negative rows within the same group.
  **Negative Rows Deduplication:** Redundant duplicate entries with negative expression type are removed.
2. Finally, if a given record is not assigned a positive-like expression type (e.g. positive, over-expression), and has a negative record, we calculate the positive rate based on the negative rate.

### Step 6: Removal of duplicates and intra studies inconsistencies

The final step of dataset preparation involved the removal of duplicate entries and the identification of intra-study inconsistencies. For this purpose, all rows for a given study were grouped by all experimental conditions, except for numeric fields (e.g. total sample count, specific sample size, specific expression percentage). Then, within each group, potential duplicates were identified using a tolerance threshold: if all rows differed by no more than 2 samples in positive sample count and by at most 3 samples in total sample count, they were considered duplicates. In such cases, only the row with the highest total sample count was retained. This tolerance was implemented to account for minor discrepancies arising from inconsistencies between different parts of the same study (e.g., abstract versus detailed tables).

If the differences between rows exceeded these thresholds, all rows in that group were excluded from the dataset. This criterion was applied under the assumption that large discrepancies reflected artificial patient subgroupings, which lacked a defined group or category during data extraction.

### Data curation summary

**Fig. 1:**
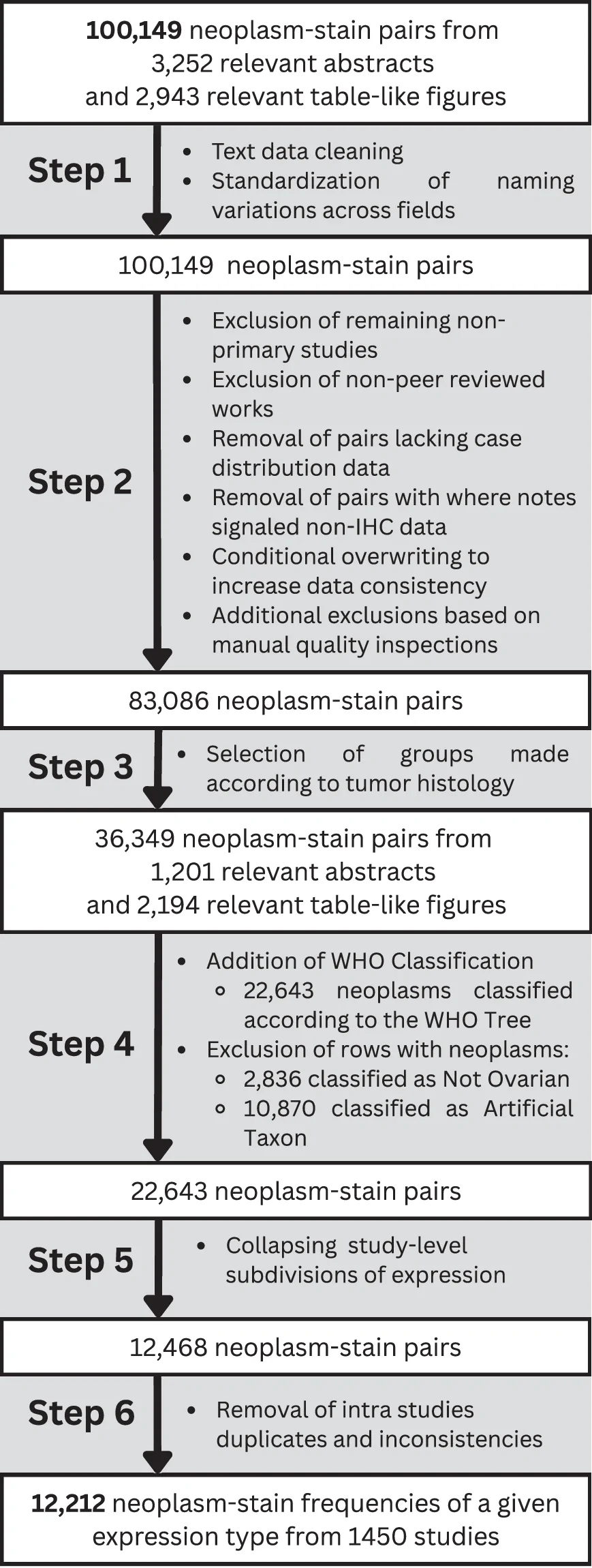
Flow-chart of the six-step post-processing pipeline and resulting database sizes.

The application of this six-step pipeline progressively reduced and refined the dataset, as summarized in Fig. 1. After text cleaning and standardization (Step 1), 100,149 tumor–stain pairs were retained. Subsequent exclusion of non-primary studies, pairs lacking case distribution data, and pairs flagged as non-IHC (Step 2) reduced the dataset to 83,086 tumor–stain pairs. Grouping according to tumor histology (Step 3) further reduced the dataset to 36,349 pairs, derived from 1,201 relevant abstracts and 2,194 table-like figures. Integration of the WHO tumor classification (Step 4) was then applied: of the 36,349 pairs, 22,643 were classified within the WHO tree and retained, while 2,836 tumors classified as “Not Ovarian” and 10,870 classified as “Artificial Taxon” were excluded. Collapsing study-level subdivisions of expression (Step 5) produced 12,468 pairs. After resolving intra-study duplicates and inconsistencies (Step 6), the final harmonized database comprised 12,212 tumor–stain frequency records of a given expression type, derived from 1,450 studies.

## Data Records

The Stainology Database is available in seven versions, corresponding to the raw extraction results and to the outputs obtained after each of the six post-processing steps (*raw.csv, step1.csv, step2.csv, step3.csv, step4.csv, step5.csv, stainology_database.csv*) as described in Data curation and harmonization. A single CSV file containing metadata for all studies retrieved from PubMed is included alongside these database versions (*papers_metadata.csv*). The dataset is available in an open Zenodo repository^14^ (https://doi.org/10.5281/zenodo.20692987).

The database is structured as a dataframe (table) in which each row represents a tumor–stain pair reported in a given study, with its associated attributes represented as columns. Table 1 lists all fields (attributes) that can be associated with a given tumor–stain pair.

**Table 1:** Description of the fields (columns) used in the database.

Field Name	Description
**Tumor (“tumor”)**	Name of the tumor.
**WHO Tumor (“matched_tumor”)**	Name of the assigned WHO tumor class or category. (e.g. class - High-grade serous carcinoma of the ovary, category - Serous tumors).
**Stain (“stain”)**	Name of the IHC stain applied (e.g., PAX-8, WT1, CK7).
**Expression Type (“expr_type”)**	Reported qualitative expression type (e.g., positive, negative, loss, over-expression, mutant).
**Total Sample Count (“total_N”)**	Total number of tissue samples analyzed for the given pair (N).
**Specific Sample Count (“expr_n”)**	Number of tissue samples showing the specified expression type (n).
**Expression Percentage (“expr_pct”)**	Percentage of tissue samples showing given expression type (%).
**Expression Score (“expr_score”)**	Assigned numerical value, which depending on the study, may refer to either the percentage of cells showcasing a specified expression type, or, more rarely, expression intensity (e.g., 0–4 or 0–4+).
**Expression Threshold (“expr_threshold”)**	Percentage of cells showcasing specified expression type (any range in the range 0–100%, e.g., >20%, 10–15%, <9%).
**Expression Intensity (“expr_intensity”)**	Expression strength (e.g., weak, moderate, high).
**Expression Distribution (“expr_distribution”)**	Staining distribution across the stained slice (e.g., focal, diffuse, block).
**Pattern/Location (“pattern”)**	Staining location within the cells or tumor tissue (e.g., cytoplasmic, membranous, nuclear, stromal).
**Group (“group”)**	Optional stratification/grouping introduced by the study (e.g., Grade, FIGO stage, age).
**Group Category (“group_cat”)**	Specific value within the group (e.g., Grade 1, FIGO Stage I).
**Notes (“notes”)**	Short notes or comments (max 5 words) generated by the LLM.
**PMID (“PMID”)**	PubMed ID (PMID) of the study.
**DOI (“doi”)**	Digital Object Identifier (DOI) of the source publication, where one exists. A small subset of records have no DOI (publications predating widespread DOI adoption, or from journals that do not assign DOIs); all such records remain identifiable via their PMID and source.
**Source (“source”)**	Citation string for the source publication, combining journal, volume, issue, pages, and date, derived from the PubMed metadata.
**Pair Source (“table_id”)**	ID of the analyzed abstract or table from the study, enabling identification of a specific abstract or table for a given study.

Both the raw and final versions of the database (the latter obtained after the sixth and final post-processing step) may contain multiple frequency entries for a given tumor–stain pair within a study. In the raw version (i.e., the pairs exactly as extracted from the literature, before any post-processing), this occurs because pairs may vary in one or more of the following fields: expression type, expression score, expression threshold, expression intensity, expression distribution, pattern/location, group, group category, and notes. Records up to and including *step2.csv* likewise preserve each study’s original stratifications (e.g., by grade, stage, or age, captured in the group and group category fields) where the source reported them. From Step 3 onward, the harmonization pipeline progressively collapses these study-specific subdivisions and aggregates the scoring, intensity, threshold, and distribution variants into a single frequency rate. As a result, in the final version multiple frequency entries may still exist for a given tumor–stain pair, but they vary only by expression type, pattern/location, and notes.

In the repository, we also provide a ground truth set of 535 extracted tumor terms manually mapped by two certified pathologists (*tumor_classification_gt.csv*) according to the *WHO Classification of tumors* (WHO Blue Book, 2020 edition) used to validate LLMs performance in the tumor WHO classification task.

## Data Overview

### Journal articles retrieval and IHC data extraction overview

A list of 1,049 unique stain names was used in search queries to compile a dataset comprising 5,961 studies. From these studies, 4,166 full-text PDFs were obtained, yielding 11,160 table-like figures. Abstracts were available for 5,910 of the 5,961 studies. Among these abstracts, 3,252 were identified as containing relevant IHC data, from which 15,216 tumor–stain pairs were extracted. For table-like figures, in the first step, 3,784 figures were identified as containing at least one neoplasm and one stain and did not meet discard criteria. In the second step, 2,943 of these figures were confirmed to include relevant IHC data, resulting in the extraction of an additional 84,933 tumor–stain pairs. A total of 100,149 tumor–stain pairs (15,216 from abstracts and 84,933 from table-like figures) were extracted from 3,657 unique studies identified as relevant from an initial pool of 5,961 screened studies.

**Fig. 2:**
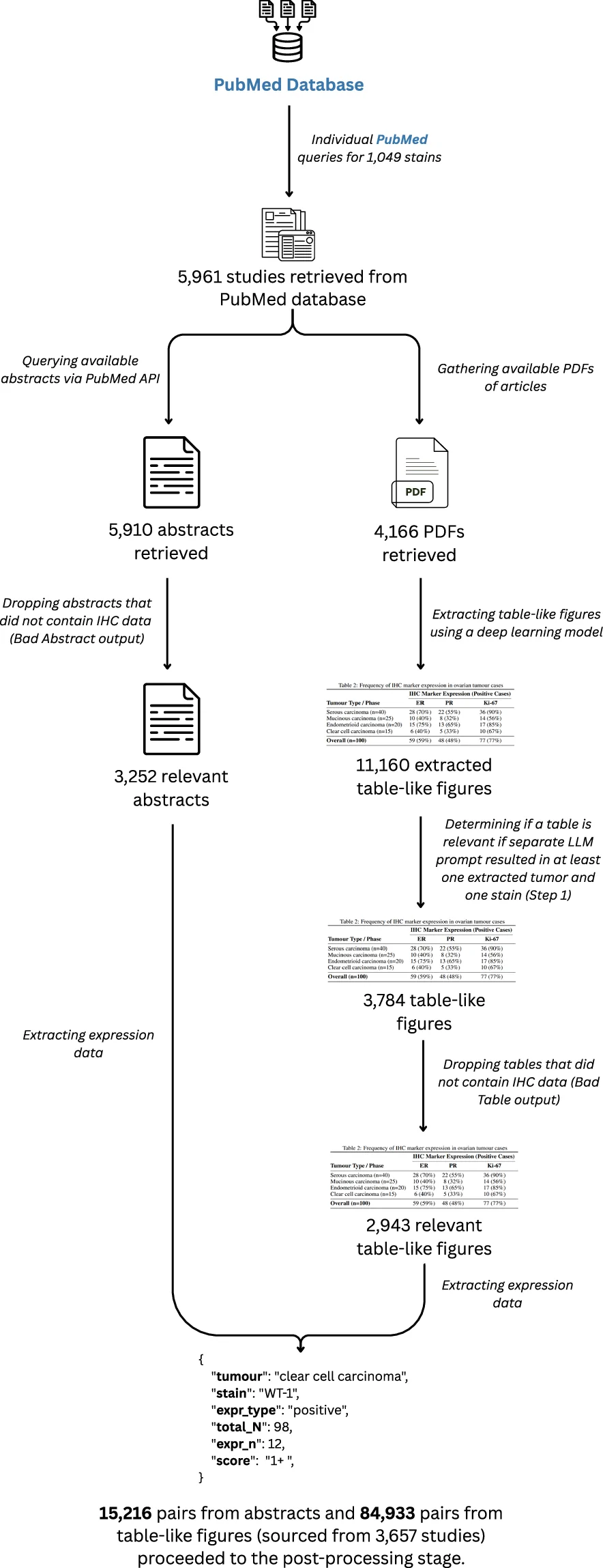
Flow chart of study retrieval and IHC data extraction results divided by phases as described in *Methods*.

A summary of these retrieval and extraction results is presented in Fig. 2.

## Technical Validation

### Validation of LLM performance in data extraction

#### Assessment of performance in identification of relevant IHC content

The study assessed whether the LLM accurately determined the presence of IHC data in abstracts and table-like figures, modeling this task as a binary classification. For abstracts, the output label “Bad Abstract”, indicating the absence of relevant data, corresponded to a negative classification. For table-like figures, a negative label was assigned if either: the LLM did not find at least one stain name and neoplasm name, or discard criteria were met during the first step of the chain; the second step yielded “Bad Table”, indicating lack of IHC data. Subsequently, a Positive label was assigned to abstract and table-like figures that contained, at least partially, IHC data.

To establish ground truth, 110 abstracts and 158 table-like figures were randomly sampled, and two pathologists independently verified whether IHC data was present, reviewing cases where their assignments differed. The sampling strategy preserved the original distribution of items with and without IHC data.

Model performance was evaluated using standard binary classification metrics, including accuracy, precision, and recall, calculated according to conventional definitions:

- Accuracy defined as the proportion of correctly classified cases among all cases divided by the total number of cases.
- Precision defined as the proportion of correctly identified positive cases among all cases predicted as positive.
- Recall defined as the proportion of true positive cases correctly identified.

In the initial classification stage, Gemini 2.5 Pro demonstrated very high efficacy in identifying the presence of relevant IHC data (Table 2). For abstract, the model only missed relevant data in 4 abstracts out of 110. On inspection, in all these cases, the abstracts reported only the total sample size without specifying case frequencies showing a given expression type. For table-like figures, only one table was misclassified. This case involved a table reporting genetic results rather than IHC. Overall, these results establish the model’s strong reliability in the first step of filtering abstracts and table-like figures for relevant IHC content.

**Table 2:** Model performance in identifying the presence of (relevant) IHC data (binary classification).

Source	Sample Size	Metric	Gemini 2.5 Pro
Abstracts	110	Accuracy	0.97
		Precision	1.00
		Recall	0.96
Table-like figures (Combined evaluation of Step 1 and Step 2 of the chain)	158	Accuracy	0.99
		Precision	0.98
		Recall	1.00

### Assessment of performance in IHC data extraction

The quality of extraction for items determined to contain IHC data was assessed. A total of 76 abstracts and 57 table-like figures were randomly sampled from the subset labeled as containing relevant data. For each example, the outputs generated by the models were reviewed by pathologists and evaluated against two binary criteria:

- Correctness defined as the condition in which all extracted tumor–stain pairs (regardless of whether all possible pairs were identified) contained entirely accurate information, exactly reflecting the content of the source.
- Completeness that required all relevant and available tumor–stain pairs and their attributes present in the abstract or table-like figure be extracted, while simultaneously meeting the correctness criterion.

These strict, binary criteria were chosen over more granular scoring methods (e.g., percentage of correctly extracted pairs) for two primary reasons: (1) simplicity in human validation and interpretation, and (2) clinical relevance—since even a single erroneous data point may influence diagnostic decision-making, only fully accurate extraction is considered acceptable.

During validation, the pathologists established the following additional rules: extracting data for pathologies other than ovarian tumors was not classified as an error; exact duplicates were not counted as errors; and instances where the model self-corrected miscalculated percentages based on reported population sizes were likewise not considered errors.

*Gemini 2.5 Pro* demonstrated consistently high extraction performance (Table 3). For abstracts, correctness was perfect, with all 76 extracted neoplasm–stain pairs accurately captured. Completeness was slightly lower (96.1%), as extraction was incomplete in 3 abstracts. In 2 of these 3 cases, the only information missed was the total sample size. For table-like figures, correctness likewise reached 100% across all 57 evaluated tables. Completeness was 94.7%, with three tables showing partially missing information. In 2 of these cases, the missing element was limited to the pattern field.

**Table 3:** Model performance in extracting neoplasm–stain pairs (quality of extraction).

Source	Sample Size	Metric	Gemini 2.5 Pro
Abstracts	76	Correctness (%)	76/76 (100%)
		Completeness (%)	73/76 (96%)
Table-like figures	57	Correctness (%)	57/57 (100%)
		Completeness (%)	54/57 (94%)

Together, these results indicate the model’s reliability in capturing relevant IHC associations, with minor omissions largely confined to secondary or descriptive fields.

Although the evaluated set showed 100% correctness, manual review during post-processing did uncover a few errors, which were manually corrected. Residual errors may still exist, but given that all 133 assessed items were correct and the few completeness gaps concerned secondary fields (e.g., sample size, pattern), any remaining issues are likely rare and of limited practical impact.

### Validation of LLM-based WHO nomenclature assignment

To support the technical quality of the LLM-assigned tumor nomenclature, the performance of the candidate LLMs was evaluated against the ground truth test set (535 terms manually mapped by two certified pathologists).

Model performance was evaluated using precision, recall, and F1-score, averaged using both macro and micro approaches.

- Precision (per-class) was defined as the fraction of instances correctly classified as belonging to that class out of all instances the model predicted to belong to that class.
- Recall (per-class) was defined as the fraction of instances in a class that the model correctly classified out of all instances in that class.
- F1-score (per-class) was defined as the harmonic mean of precision and recall.
- Macro-averaged metrics were the unweighted mean of per-class values, giving equal weight to each class.
- Micro-averaged metrics were calculated by aggregating the true positives, false positives, and false negatives for all classes, effectively weighting the metric by class frequency.

The custom categories “Artificial Taxon” and “Not Ovarian” were excluded from the metric calculations, as they were out of scope for classification performance. Precision was also separately calculated on the subset of predictions *excluding* these two categories to assess performance on terms intended for the final database.

All tested models showed high performance (Table 4). The Google Gemini 2.5 Flash model achieved the highest macro-averaged metrics (F1-score: 0.89; Recall: 0.91) and a micro-averaged F1-score of 0.96, matching the GPT-5 model. Consequently, Gemini 2.5 Flash was selected for the final classification task.

**Table 4:** Classification performance of LLMs in assignment of extracted tumor names according to the *WHO Classification of tumors* (WHO Blue Book, 2020 edition) evaluated on a test set consisting of 535 elements.

Model	Precision (micro/macro)	Precision excl. Not Ovarian/Artificial Taxon predictions (micro/macro)	Recall (micro/macro)	F1-score (micro/macro)
GPT-5 Mini	0.90/0.72	0.94/0.76	0.87/0.77	0.88/0.73
GPT-5	0.96/0.87	0.98/0.88	0.95/0.86	0.96/0.85
Gemini Pro 2.5	0.96/0.84	0.97/0.85	0.91/0.84	0.93/0.83
Gemini Flash 2.5	0.95/0.88	0.98/0.91	0.96/0.91	0.96/0.89

For the selected Gemini 2.5 Flash model, the micro-precision (excluding “Not Ovarian” and “Artificial Taxon” predictions) was **0.98**. This corresponded to only 8 misclassifications out of 353 terms that would be included in the database. A manual inspection of said 8 misclassified entries for Gemini Flash 2.5 revealed that all errors consisted of assigning a specific class within the correct family of tumors (e.g., “Clear cell carcinoma of the ovary” prediction versus “Malignant Clear Cell tumors” ground truth).

## Usage Notes

The project team welcomes community contributions. Researchers who wish to submit a relevant ovarian IHC study that is not included, or is only partially represented (e.g., extracted from an abstract alone), are encouraged to contact the project team through the project website (https://stainology.cs.put.poznan.pl); the full-text PDF and the corresponding PubMed ID (PMID) are sufficient for a study to be considered. In addition to the repository deposit, the dataset can also be downloaded and filtered at the same address.

The post-processing pipeline is implemented as a set of modular classes, each responsible for a specific function, which are combined into a workflow (pipeline). The design allows the pipeline to be easily adapted or extended to meet specific analytical needs. The source code details can be found in Code Availability.

Intended use: The database reports IHC expression data as published in the source literature and is not a curated clinical-interpretation guideline. It is designed to support research, meta-analyses, and computational model development; clinical decision-support applications are a future objective conditioned on further validation.

Pattern-based markers and legacy terminology: For markers with pattern-dependent interpretation (including p53, Rb, ARID1A, mismatch-repair proteins, and β-catenin), older publications frequently used generic labels such as “positive”/“negative” that predate and may not correspond to current conventions (e.g., “wild-type”, “abnormal”, “mutant”). Source terminology is preserved verbatim; users should account for terminological evolution by restricting records by publication date or applying appropriate term mappings where applicable.

Precision was prioritized over sensitivity. During article retrieval, studies focused on reclassification were excluded to prevent errors arising from the difficulty of distinguishing primary from reclassified frequency records. Similarly, a portion of extracted data that could not be harmonized during post-processing was excluded, which inevitably reduced the overall volume of retained data.

## Data Availability

The dataset is available in an open Zenodo^14^ repository (https://doi.org/10.5281/zenodo.20692987). The repository deposit includes all nine CSV files: the seven versions of the main database (from the raw extraction, *raw.csv*, through to the final harmonized *stainology_database.csv*), the articles metadata file (*papers_metadata.csv*), and the ground truth tumor classification file (*tumor_classification_gt.csv*). In addition, a *README.md* file containing a description of the data fields is provided.

## Code availability

The full source code used to create and post-process the database is available in the public GitHub repository: https://github.com/jan-gruszczynski-publications/stainology-paper and is archived under the https://doi.org/10.5281/zenodo.17635328.

The repository includes all scripts used to retrieve relevant scientific articles, identify relevant abstracts and tables, extract IHC data, and perform the complete post-processing pipeline used to create the final version of the database.
